# Physical activity and the brain myelin content in humans

**DOI:** 10.3389/fncel.2023.1198657

**Published:** 2023-06-05

**Authors:** Mariusz J. Kujawa, Anna B. Marcinkowska, Małgorzata Grzywińska, Monika Waśkow, Alexander Romanowski, Edyta Szurowska, Paweł J. Winklewski, Arkadiusz Szarmach

**Affiliations:** ^1^2nd Department of Radiology, Medical University of Gdańsk, Gdańsk, Poland; ^2^Applied Cognitive Neuroscience Lab, Department of Neurophysiology, Neuropsychology and Neuroinformatics, Medical University of Gdańsk, Gdańsk, Poland; ^3^Neuroinformatics and Artificial Intelligence Lab, Department of Neurophysiology, Neuropsychology and Neuroinformatics, Medical University of Gdańsk, Gdańsk, Poland; ^4^Institute of Health Sciences, Pomeranian University in Słupsk, Słupsk, Poland; ^5^Department of Psychiatry and Psychotherapy, Helios Klinik, Hettstedt, Germany; ^6^Department of Neurophysiology, Neuropsychology and Neuroinformatics, Medical University of Gdańsk, Gdańsk, Poland

**Keywords:** myelin, magnetic resonance imaging (MRI), human brain, physical activity, myelination and remyelination

## Abstract

New imaging sequences and biophysical models allow adopting magnetic resonance imaging (MRI) for *in vivo* myelin mapping in humans. Understanding myelination and remyelination processes in the brain is fundamental from the perspective of proper design of physical exercise and rehabilitation schemes that aim to slow down demyelination in the aging population and to induce remyelination in patients with neurodegenerative diseases. Therefore, in this review we strive to provide a state-of-the art summary of the existing MRI studies in humans focused on the effects of physical activity on myelination/remyelination. We present and discuss four cross-sectional and four longitudinal studies and one case report. Physical activity and an active lifestyle have a beneficial effect on the myelin content in humans. Myelin expansion can be induced in humans throughout the entire lifespan by intensive aerobic exercise. Additional research is needed to determine (1) what exercise intensity (and cognitive novelty, which is embedded in the exercise scheme) is the most beneficial for patients with neurodegenerative diseases, (2) the relationship between cardiorespiratory fitness and myelination, and (3) how exercise-induced myelination affect cognitive abilities.

## 1. Introduction

New imaging sequences and biophysical models hold promise for the ability to adopt magnetic resonance imaging (MRI) for *in vivo* myelin mapping in humans. Hopefully, MRI will allow for large, longitudinal, painless and non-invasive studies focused on tracking of the cellular composition of neuronal and extra-neuronal brain tissue (Paquola and Hong, 2023). The mean correlation between MRI and histology is good, with *R*^2^ = 0.54 [standard deviation (SD) = 0.30] for animal studies, and *R*^2^ = 0.54 (SD = 0.18) for human studies. Moreover, the reproducibility for the various MRI methods seems acceptable [interclass correlation coefficient (ICC) = 0.75–0.93, *R*^2^ = 0.90–0.98, coefficient of variance = 1.3–27%], except for the magnetization transfer ratio (ICC = 0.05–0.51) (van der Weijden et al., 2021). Due to its three-dimensional nature, MRI offers the potential ability to visualize myeloarchitecture in three dimensions, along with biologically meaningful anatomical axes, compared with two-dimensional scans offered by classical histology. Finally, MRI delivers the unique possibility to assess the contribution of region-specific myelination to large-scale organization, offering a tool to unveil how myelination affects global brain networks (Paquola and Hong, 2023).

Myelin is a lipid-rich membrane that sheathes axons, speeds up information transfer embodied in action potentials, provides protection and metabolic support to covered neurons, and is indispensable for the proper cognitive functioning and motor tasks undertaken by the nervous system (Saab and Nave, 2017; Bechler et al., 2018; Elazar et al., 2019). Myelination development over the lifespan resembles an inverted U-shape curve, with the highest brain myelin content from 30 to 60 years of age, and subsequent decline after 60 years of age (Naumburg et al., 2012; Grydeland et al., 2013; Aviv et al., 2014; Bouhrara et al., 2021; Sui et al., 2022). Demyelination is at the core of neurodegenerative disorders and cognitive decline in physiological aging. It is increasingly recognized that abnormalities associated with the brain myelin content are not limited to information transfer speed within individual axons; they also affect the synchrony of network circuits (Bartzokis, 2004, 2005). Consequently, there is a constant search for novel pharmacological and non-pharmacological interventions to slow down and possibly revert demyelination processes.

Animal studies provide a quite complex scheme with regard to myelination and remyelination. There are two terms that have been coined to describe the underlying mechanisms behind myelination physiology and pathophysiology: adaptive myelination and remyelination (Bloom et al., 2022). Adaptive myelination refers to the expansion of already existing myelin sheaths or the addition of supplementary myelin to myelinated brain regions. Remyelination is understood as the covering of demyelinated axons with a new myelin (Bloom et al., 2022). Adaptive myelination is triggered by novel learning experiences—for example, when an animal acquires a novel motor task (McKenzie et al., 2014; Zhou et al., 2016). It is believed that the first 24 h following exposure to novel motor tasks are critical and actually define the opportunity window for adaptive myelination (Bloom et al., 2022). It remains unclear if, in animals, voluntary exercise not associated with novel learning may result in adaptive myelination. In contrast, remyelination is distinct from adaptive myelination and might be induced by voluntary exercise not associated with novel motor task learning (Bloom et al., 2022).

It remains unknown if the same scheme applies to humans. In their excellent review, Bloom et al. (2022) suggest that such direct translation may exist. Nevertheless, to support their reasoning, they cite only diffusion tensor imaging (DTI) studies in humans, referring to fractional anisotropy as a main marker of white matter integrity. Fractional anisotropy, however, is a marker that reflects the entire complexity of the neuronal tissue microstructure (Jones and Cercignani, 2010; Winklewski et al., 2018). Consequently, it is likely that fractional anisotropy does not detect subtle changes in myelination. The topic is of fundamental significance from the perspective of proper design of physical exercise and rehabilitation schemes to slow down demyelination in the aging population and to induce remyelination in patients with neurodegenerative diseases.

Therefore, in this review we strived to provide a state-of-the art summary of the existing MRI studies in humans focused on the effects of physical activity on myelination/remyelination. Electronic databases PubMed and Scopus were searched for relevant studies; last search was performed on 23 March 2023. The reference lists of included studies were hand-searched for additional references. A two-step approach was used to select articles. Firstly, titles and abstracts of all search results were screened for the following characteristics (1) original article and (2) published in English. Secondly, full-text articles were obtained from the selected studies and were reviewed on the following inclusion criteria (1) performed physical activity intervention and (2) performed MRI assessment of myelin. Two researchers independently reviewed titles, abstracts, full texts and extracted data. The following search terms were used: myelin AND exercise OR physical activity AND MRI. To date, there are only nine publications in this field (four cross-sectional studies, four longitudinal studies, and one case report; Table 1). Nevertheless, some initials conclusions can be drawn and recommendations for future direction in the research in this area may be formulated.

**TABLE 1:** Summary of MRI studies investigating the brain myelin content in humans.

References	Study design	MRI sequences	Conclusion
Boa Sorte Silva et al., 2023	Cross-sectional study: older adults with cerebral small vessel disease and mild cognitive impairment	The myelin water fraction was defined as the fraction of signal with T2 < 40 ms over the entire T2 distribution	Greater physical activity was linked to a higher myelin content in the whole-brain white matter, in the sagittal stratum, the anterior corona radiata and the genu of the corpus callosum
Kirby et al., 2022	Longitudinal study: 2-week motor-training in healthy subjects	The water myelin fraction was obtained using a 32 echo gradient and spin echo (GRASE) sequence T2 scan	Higher myelin content induced by physical exercise augments axonal transmission efficiency in the cortico-spinal tract
Greeley et al., 2022	Cross-sectional study: chronic stroke (>6 months) patients vs. older healthy controls	The water myelin content was defined as the sum of the amplitudes within a short T2 signal (15–40 ms) divided by the sum of the amplitudes for the total T2 distribution	More physically active patients showed less myelin asymmetry in brain regions responsible for movement
Shao et al., 2022	Cross-sectional study: elite golf players compared with control participants	Maps of macromolecular tissue volume and quantitative T1 for each participant were generated from spoiled gradient echo images and spin-echo inversion recovery images	Golf players showed higher myelin content in the left temporal pole than non-players
Tobiume et al., 2022	Case study: an alcohol-dependent man who recovered from central pontine myelinolysis	Abnormality detected in the central pons in diffusion-weighted images as a relatively high-signal area resembling a piglet sign but not clear in T2- and T1-weighted images, 1.5 T MRI	Physical exercise therapy is important for improving the prognosis of central pontine myelinolysis
Mendez Colmenares et al., 2021	Longitudinal study: 6-month aerobic walking and dancing interventions in older adults	Calibrated T1- to T2-weighted images	White matter plasticity is induced by aerobic walking and dance in the late myelinating regions containing association and commissural fibers: the genu and the splenium of the corpus callosum, the forceps minor and the cingulum
Rowley et al., 2020	Longitudinal study: 12 weeks of exercise three times a week on a stationary bike in healthy subjects	Quantitative T1 and proton density maps with B1 correction for cortical segmentation and R1 images calculated as R1 = 1/T1	Exercise may upregulate myelination in the motor regions of the cerebral cortex
Casella et al., 2020	Longitudinal study: 2 months of drumming training in patients with Huntington disease	Changes in white matter microstructure were investigated with DTI-based metrics, the restricted diffusion signal fraction from the composite hindered and restricted model of diffusion and the macromolecular proton fraction from quantitative magnetization transfer imaging	Significantly greater change in the macromolecular proton fraction in patients with Huntington’s disease relative to controls in the CCII and CCIII segments of the corpus callosum, and the right white matter pathways linking the putamen and the supplementary motor areas post-training
Bracht et al., 2016	Cross-sectional study: young healthy subjects	Multicomponent driven equilibrium single pulse observation of T1 and T2	Higher physical activity is associated with greater myelination of the right parahippocampal cingulum

## 2. Cross-sectional studies

Bracht et al. (2016) recruited 33 healthy participants (19 women, 14 men; 25.5 ± 4.2 years of age). All volunteers were right handed and were either undergoing or had previously completed a university degree course. They excluded professional athletes, musicians and subjects involved in amateur sport at competitive levels. The authors used multicomponent driven equilibrium single pulse observation of T1 and T2 (McDESPOT) to visualize whole brain estimated myelin water fraction maps. They found a selective positive correlation between activity level and myelin water fraction in the right parahippocampal cingulum (*r* = 0.482, *p* = 0.007). Importantly, they found no correlations between DTI fractional anisotropy and physical activity. Therefore, the authors concluded that the water myelin fraction “not only represents a more specific marker for myelination but is also a more sensitive marker than fractional anisotropy for detecting associations between white matter microstructure and physical activity.”

Greeley et al. (2022) investigated patients with chronic stroke (>6 months) and matched older healthy controls (the ages from 40 to 85 years). Myelin asymmetry ratios, calculated as a ratio of contralesional to ipsilesional myelin water fraction was analyzed in patients and healthy volunteers with high and low level of physical activity. Physical activity was assessed using accelerometers fixed on the subjects’ wrists for 3 consecutive days (72 h). The water myelin content was defined as the sum of the amplitudes within a short T2 signal (15–40 ms) divided by the sum of the amplitudes for the total T2 distribution. Asymmetries in water myelin content were investigated in the following motor regions of interest (chosen *a priori*): superior corona radiata, posterior corona radiata, cerebral peduncle, anterior limb of internal capsule and posterior limb of the internal capsule. Patients in the high physical activity stroke group had myelin content asymmetry comparable to that noted in older healthy adults. In contrast, the low physical activity stroke patients exhibited greater myelin content asymmetry compared to older healthy controls. Moreover, myelin content in the anterior limb of internal capsule, posterior corona radiata and superior corona radiata was positively linked to wrist movement activity across all participants.

Shao et al. (2022) included 55 adults: 28 golf players (20 men and 8 women; 24.61 ± 5.20 years old) and 27 non-players (19 men and 8 women; 24.56 ± 4.68 years old). All the participants were right handed, physically and neurologically healthy with normal or corrected-to-normal vision. The authors generated maps of macromolecular tissue volume and quantitative T1 for each participant from spoiled gradient echo images and spin-echo inversion recovery images. They processed the obtained images by using the mrQ software package.^1^ They found augmented myelin content in the left temporal pole in elite golf players compared with the non-players. Importantly, they found that increased microstructural plasticity was positively linked to golfing proficiency.

Boa Sorte Silva et al. (2023) enrolled 102 community-dwelling older adults (63.7% women; 74.7 ± 5.5 years old) with cerebral small vessel disease (diagnosed with MRI), and mild cognitive impairment defined as scoring <26 on the Montreal Cognitive Assessment. None of participants had a significant functional impairment or a prior diagnosis of dementia. They calculated the myelin water fraction as the fraction of signal with T2 < 40 ms over the entire T2 distribution. Then, they developed voxel-wise myelin water fraction maps for each participant and extracted data for whole-brain and tract-specific white matter. They found that active lifestyle (including physical activity) is associated with a higher myelin water fraction in the whole-brain white matter and in the following tracts: the anterior corona radiata, the genu of the corpus callosum and the sagittal stratum. Because the associations were independent of age, sex, body mass index, education and white matter volume, the authors concluded that physical activity may slow down myelin loss in adults with cerebral small vessel disease and mild cognitive impairment.

To conclude, the cross-sectional studies have demonstrated that more physically active adults have better myeloarchitecture profiles compared with less active adults. Importantly, this association remains valid throughout the entire adult lifespan, from the early 20 s to past the 70 s, in men and women. High physical activity profile seems to be neuroprotective in stroke patients. The authors used various MRI modalities to visualize the brain myelin content while the association between physical activity and estimated myelination remained clearly visible.

## 3. Longitudinal studies

Casella et al. (2020) investigated eight patients with Huntington’s disease (48.5 ± 15.62 years old, range 22–68 years) and nine matched controls (52.6 ± 14.56 years old, range 22–68 years). Six patients were at an early stage of the disease and two were at a more advanced stage. The participants performed drumming training: 15 min per day, 5 times per week, for 2 months. The drumming pattern was based on one of the following rhythms: Brazilian samba, Spanish rumba, West-African kuku and Cuban son (Metzler-Baddeley et al., 2014). The authors investigated changes in the white matter microstructure with DTI-based metrics, the fraction of restricted diffusion from the composite hindered and restricted model of diffusion, and the macromolecular proton fraction from quantitative magnetization transfer imaging. The drumming exercise resulted in significantly higher training-induced macromolecular proton fraction (myelin plasticity marker) changes within the corpus callosum (CCII and CCIII) and the pathways linking the right supplementary motor areas and the putamen in patients with Huntington’s disease compared with controls. Myelin plasticity did not change in the control group. The authors described the macromolecular proton fraction changes in patients relative to controls as a “catch-up effect to the better baseline status of the control group.”

Rowley et al. (2020) evaluated MRI scans from 47 healthy subjects: 22 in the passive control group (9 women; 72.8 ± 5.4 years old) and 25 in exercise group (15 women; 76.8 ± 8.0 years old). The exercise intervention comprised 12 weeks of supervised cycle ergometer training with three 30-min sessions per week. They visualized changes by examining myelin quantitative T1 and proton density maps with B1 correction for cortical segmentation and R1 images calculated as R1 = 1/T1. There was a significant difference in R1 between the groups, with R1 augmentation in the cycling group in the leg region of the motor cortex. The authors indicated that “aerobic cycling exercise promotes the maintenance and growth of the myelin sheaths in this region in the elderly.” In the control group, they reported a correlation between ventilatory threshold (equivalent to 64 ± 9% VO_2_max) and myelin content. Importantly, the Rowley et al. (2020) publication is the only one describing the longitudinal study focused on cortical myelination.

Mendez Colmenares et al. (2021) investigated 180 healthy older adults in three groups: control (43 participants, 26 women; 66.3 ± 4.5 years old), dance (51 participants, 37 women; 65.8 years ± 4.6 years old) and walking (86 participants, 54 women; 64.8 ± 4.2 years old). The active control consisted of exercises lasting 24 weeks and aiming at improvement in flexibility, strength and balance and involved some yoga mats and blocks, chairs and resistance bands. All exercised were adjusted for individuals ≥60 years old. The walking intervention aimed at augmenting cardiorespiratory fitness. It started with walking sessions at 50–60% of the maximal heart rate, then the walking duration was prolonged from 20 to 40 min during the first 6 weeks of the programme. During the last 18 weeks, the volunteers walked during each session for 40 min at 60–75% of their maximal heart rate. The dance intervention delivered combined cognitive and social enrichment associated with aerobic physical exercise. The dance choreography was increasingly challenging during the 6-month course. The authors calculated the T1-/T2-weighted (T1W/T2W) ratio from calibrated T1- to T2-weighted images to detect white matter integrity/myelin content. The effects of the aerobic exercise (both walking and dancing) on the T1W/T2W signal was significant for the mean of all white matter voxels. Moreover, regional analyses indicated a specific effect in the late myelinating regions containing association and commissural fibers: the genu and splenium of the corpus callosum, the forceps minor and the cingulum. There were no associations between augmented cardiorespiratory fitness and change in the T1W/T2W signal. This signal diminished during the 6-month period in the majority of white matter brain regions in the active control group.

Kirby et al. (2022) enrolled twelve healthy, right-handed subjects (seven women) who performed a 2-week motor-training schedule consisting of a visual-motor maze task with significant level of difficulty. Both non-dominant and dominant hands were trained and examined. There was no control group. The volunteers were young adults: mean age of 25.8 ± 3.7 years. The water myelin fraction was obtained using a 32 echo gradient and spin echo (GRASE) sequence T2 scan. Myelin water fraction was defined as the fraction of the geometric mean T2 distribution with 10 ms < T2 < 25 ms over the total geometric mean T2 distribution. The authors associated the water myelin content with structural (DTI fractional anisotropy) and functional (blood oxygen level dependent signal low frequency oscillations) to conclude that the higher myelin content the better axonal transmission in corticospinal tract.

In summary, the longitudinal studies have clearly shown that intense aerobic exercise (such as walking, cycling or dancing) can induce myelination in specific regions associated with particular activity (i.e., motor cortex) or late myelinating regions that are particularly vulnerable to demyelination with aging. Exposure to physical training of high complexity seems to result in higher myelin content and improved axonal transmission. There seems to be no relation between favorable changes in cardiorespiratory fitness evoked by intense aerobic exercise and myelin expansion. The link between aerobic fitness and myelin content is seen sometimes in control groups (so a similar finding like in cross-over studies). Physical activity of low intensity (such as drumming or exercise designed to improve flexibility, strength and balance) did not produce a favorable effect (in terms of myelin content augmentation) in healthy subjects. Nevertheless, drumming appeared to be very effective in remyelination in patients with Huntington disease.

## 4. Case report

Central pontine myelinolysis is a rare neurological complication that occurs after rapid correction of hyponatremia in patients with malnutrition, alcoholism and severe burns. The main pathophysiological process is symmetric demyelination of the central pons (Singh et al., 2014). A 44-year-old man with a past history of chronic alcoholism had an abnormality typical for central pontine myelinolysis in the central pons detected on axial MRI (high-signal area resembling a piglet sign in diffusion-weighted images; not clear in T2W and T1W images). The central pontine piglet sign was clearly defined in T2W and T1W images 11 weeks after disease onset. The diagnosis was based on radiological examination (MRI) and clinical signs and symptoms (Tobiume et al., 2022). Rehabilitation was not particularly specific or sophisticated, as no specialized equipment was used (commonly available aids such as a chair, parallel bars, a walker and a cane were applied), but it was regular (about 1 h daily). The patient was completely paraplegic on admission (but with contact, could express “Yes” or “No” either by blinking or moving his eyes). After half a year, the patient was discharged with only slightly slurred speech and minor muscle weakness in his right hand. Unfortunately, the remyelination process was not confirmed by MRI examination. Consequently, we can only infer from clinical picture that such process most likely occurred.

## 5. Discussion

Based on the available literature, it seems that two relatively firm conclusions can be made: (1) an active lifestyle is associated with augmented myelin content in the human brain and (2) with the use of intense aerobic exercise myelin expansion can be achieved in healthy subjects throughout the entire adult lifespan. One small study in patients with Huntington’s disease (Casella et al., 2020) and a case report describing a subject with central pontine myelinolysis (Tobiume et al., 2022) may suggest that even not very intensive but regular physical activity can result in remyelination in the brain areas affected by demyelination. Active life style also seems to be neuroprotective in stroke patients (Greeley et al., 2022). However, this very positive finding needs to be confirmed in larger studies. Less intense physical activity could represent a very valuable option for patients with neurodegenerative diseases associated with demyelination. It is quite surprising that to date, there is no study in patients with multiple sclerosis.

Less intense physical activity does not seem to cause myelin expansion in healthy subjects (Casella et al., 2020; Mendez Colmenares et al., 2021). This is an interesting finding and suggests that in humans as in animals (rodents) there are two different patterns with regard to myelination and remyelination processes (Bloom et al., 2022). Overall, such a pattern is consistent with the wider consensus that exercise intensity to induce neuronal plasticity can be scalable in healthy individuals but not necessarily in patients with neurological conditions (Hortobágyi et al., 2022). Additional research is needed to determine how humans (in terms of myelin content) respond to physical exercise associated with novelty. Kirby et al. (2022) study may suggest that physical training of high complexity augments myelination and improve axonal transmission in involved brain structures.

The relationship between cardiorespiratory fitness and myelination remains unclear. An active lifestyle is usually associated with better cardiorespiratory performance (Castells-Sánchez et al., 2021). However, the relationship between the cardiorespiratory profile and brain myelin content has not yet been investigated in cross-sectional studies. The only longitudinal study where the authors considered this relationship showed no relation between favorable changes in cardiorespiratory fitness evoked by intense aerobic exercise and myelin expansion (Mendez Colmenares et al., 2021). This finding is somewhat contradictory to the belief that exercise-induced changes in cardiovascular performance represent a key element in cognitive improvement (Aczel et al., 2022; Maleki et al., 2022; Renke et al., 2022).

The main weakness of the presented studies is that all of them, except of Rowley et al. (2020), refer to myelin content in white matter. The latest study by Sandrone et al. (2023) comparing MRI T1W/T2 sequences with *ex vivo* post-mortem histology suggests that MRI T1W/T2W sequences correlate well with myelin content in the cortical gray matter, but may not be a specific method to map myelin density in the callosal white matter. This limitation particularly refers to Bracht et al. (2016) and Mendez Colmenares et al. (2021) studies. In contrast, T1 or 1/T1 is very related to brain water content (Lee et al., 2021; Paquola and Hong, 2023). Brain water content, in turn, is affected by pathophysiological processes such as neuroinflammation or edema which generally makes white matter integrity assessment in MRI settings more complex (Winklewski et al., 2018). Nevertheless, in the reviewed studies the T1 and 1/T1 sequences were used in young healthy subjects (Rowley et al., 2020; Shao et al., 2022).

It should be emphasized that the authors of the reviewed articles used various MRI techniques, including MTV (Myelin Volume Fraction), MWF (Myelin Water Fraction), mcDESPOT MWF (Multi-Component Driven Equilibrium Single Pulse Observation of T1 and T2 with Myelin Water Fraction), and MPF (Magnetization Transfer Ratio Pool Size Ratio). These are all practical MRI techniques for quantifying tissue properties but have some limitations (Lee et al., 2021; Zhou et al., 2023). Firstly, these techniques are limited by their spatial resolution, making it difficult to accurately measure tissue properties in small structures or regions of interest. It can be particularly challenging for techniques like mcDESPOT MWF, which require high spatial resolution to accurately distinguish between different tissue compartments (Deoni and Kolind, 2015). Secondly, the accuracy and reproducibility of MRI techniques can be highly dependent on the specific acquisition parameters used, such as the magnetic field strength, sequence type, and imaging parameters. It can create difficulties in comparing results across different studies or institutions (Lee et al., 2021). Thirdly, MRI signal is inherently noisy, limiting the accuracy and precision of quantitative measurements. Techniques like mcDESPOT MWF, which require multiple scans with different acquisition parameters, can be susceptible to noise (Alonso-Ortiz et al., 2018). Additionally, tissue properties can vary widely between individuals, making it problematic to establish normative values or detect subtle changes in disease states. It can pose significant difficulties for techniques like MPF, which rely on comparing different tissue compartments (Khodanovich et al., 2017). While these techniques are valuable tools for studying tissue microstructure, they should be interpreted cautiously and in the context of other clinical and imaging data (van der Weijden et al., 2023).

Finally, future research should examine how changes in myelin content evoked by physical activity translate into cognitive improvement in patients and healthy subjects. Thus, extensive neuropsychological testing should become a gold standard particularly in longitudinal studies.

## 6. Conclusion

Physical activity and an active lifestyle have a beneficial effect on the myelin content in humans. Myelin expansion can be induced in humans throughout the entire lifespan by intense aerobic exercise. Additional research is needed to determine what exercise intensity (and embedded in exercise scheme cognitive novelty) is the most beneficial for patients with neurodegenerative diseases. The relationship between cardiorespiratory fitness and myelination has yet to be established. Extensive neuropsychological testing is needed to determine how exercise-induced myelination affects cognitive abilities.

## Author contributions

MK, AM, MG, PW, and AS: conceptualization. MK: methodology, investigation, writing—original draft preparation, and project administration. AM, MG, MW, AR, ES, PW, and AS: validation and supervision. MK, AM, and MG: formal analysis. ES, PW, and AS: resources. MK, ES, and AS: funding acquisition. All author: writing—review and editing, read, and agreed to the published version of the manuscript.

## Footnotes

1
https://github.com/mezera/mrQ

## References

[B1] Aczel D. Gyorgy B. Bakonyi P. BukhAri R. Pinho R. Boldogh I. (2022). The systemic effects of exercise on the systemic effects of Alzheimer’s disease. *Antioxidants* 11:1028. 10.3390/ANTIOX11051028 35624892 PMC9137920

[B2] Alonso-Ortiz E. Levesque I. Pike G. (2018). Multi-gradient-echo myelin water fraction imaging: comparison to the multi-echo-spin-echo technique. *Magn. Reson. Med.* 79 1439–1446. 10.1002/MRM.26809 28656649

[B3] Aviv M. Yeatman J. Stikov N. Kay K. Cho N. Dougherty R. (2014). Quantifying the local tissue volume and composition in individual brains with MRI. *Pediat. Nephrol.* 29 1231–1238. 10.14440/jbm.2015.54.A 24185694 PMC3855886

[B4] Bartzokis G. (2004). Age-related myelin breakdown: a developmental model of cognitive decline and Alzheimer’s disease. *Neurobiol. Aging* 25 5–18. 10.1016/j.neurobiolaging.2003.03.001 14675724

[B5] Bartzokis G. (2005). Brain myelination in prevalent neuropsychiatric developmental disorders: primary and comorbid addiction. *Adoles. Psychiatry* 29 55–96. 18668184 PMC2490819

[B6] Bechler M. Swire M. Ffrench-Constant C. (2018). Intrinsic and adaptive myelination-a sequential mechanism for smart wiring in the brain. *Dev. Neurobiol.* 78 68–79. 10.1002/DNEU.22518 28834358 PMC5813148

[B7] Bloom M. Orthmann-Murphy J. Grinspan J. (2022). Motor learning and physical exercise in adaptive myelination and remyelination. *ASN Neuro* 14:17590914221097510. 10.1177/17590914221097510 35635130 PMC9158406

[B8] Boa Sorte Silva N. Dao E. Liang H. C. Tam R. Lam K. Alkeridy W. (2023). Myelin and physical activity in older adults with cerebral small vessel disease and mild cognitive impairment. *J. Gerontol. Ser. A Biol. Sci. Med. Sci.* 78 545–553. 10.1093/GERONA/GLAC149 35876839

[B9] Bouhrara M. Kim R. Khattar N. Qian W. Bergeron C. Melvin D. (2021). Age-related estimates of aggregate G-ratio of white matter structures assessed using quantitative magnetic resonance neuroimaging. *Hum. Brain Mapp.* 42 2362–2373. 10.1002/HBM.25372 33595168 PMC8090765

[B10] Bracht T. Jones D. Bells S. Walther S. Drakesmith M. Linden D. (2016). Myelination of the right parahippocampal cingulum is associated with physical activity in young healthy adults. *Brain Struct. Funct.* 221 4537–4548. 10.1007/S00429-016-1183-3 26786737 PMC5102942

[B11] Casella C. Bourbon-Teles J. Bells S. Coulthard E. Parker G. Rosser A. (2020). Drumming motor sequence training induces apparent myelin remodelling in huntington’s disease: a longitudinal diffusion MRI and quantitative magnetization transfer study. *J. Huntington’s Dis.* 9 303–320. 10.3233/JHD-200424 32894249 PMC7836062

[B12] Castells-Sánchez A. Roig-Coll F. Dacosta-Aguayo R. Lamonja-Vicente N. Sawicka A. Torán-Monserrat P. (2021). Exercise and fitness neuroprotective effects: molecular, brain volume and psychological correlates and their mediating role in healthy late-middle-aged women and men. *Front. Aging Neurosci.* 13:615247. 10.3389/FNAGI.2021.615247 33776741 PMC7989549

[B13] Deoni S. Kolind S. (2015). Investigating the stability of McDESPOT myelin water fraction values derived using a stochastic region contraction approach. *Magn. Reson. Med.* 73 161–169. 10.1002/MRM.25108 24464472 PMC8487011

[B14] Elazar N. Vainshtein A. Rechav K. Tsoory M. Eshed-Eisenbach Y. Peles E. (2019). Coordinated internodal and paranodal adhesion controls accurate myelination by oligodendrocytes. *J. Cell Biol.* 218 2887–2895. 10.1083/JCB.201906099 31451613 PMC6719437

[B15] Greeley B. Rubino C. Denyer R. Chau B. Larssen B. Lakhani B. (2022). Individuals with higher levels of physical activity after stroke show comparable patterns of myelin to healthy older adults. *Neurorehabil. Neural Repair* 36 381–389. 10.1177/15459683221100497 35533214 PMC9127936

[B16] Grydeland H. Walhovd K. B. Tamnes C. K. Westlye L. T. Fjell A. M. (2013). Intracortical myelin links with performance variability across the human lifespan: results from T1- and T2-weighted MRI myelin mapping and diffusion tensor imaging. *J. Neurosci.* 33 18618–18630. 10.1523/JNEUROSCI.2811-13.2013 24259583 PMC6618798

[B17] Hortobágyi T. Vetrovsky T. Balbim G. Sorte Silva N. Manca A. Deriu F. (2022). The impact of aerobic and resistance training intensity on markers of neuroplasticity in health and disease. *Ageing Res. Rev.* 80 18618–18630. 10.1016/J.ARR.2022.101698 35853549

[B18] Jones D. Cercignani M. (2010). Twenty-five pitfalls in the analysis of diffusion MRI data. *NMR Biomed.* 23 803–820. 10.1002/NBM.1543 20886566

[B19] Khodanovich M. Sorokina I. Glazacheva V. Akulov A. Nemirovich-Danchenko N. Romashchenko A. (2017). Histological validation of fast macromolecular proton fraction mapping as a quantitative myelin imaging method in the cuprizone demyelination model. *Sci. Rep.* 7 1–12. 10.1038/srep46686 28436460 PMC5402392

[B20] Kirby E. Frizzell T. Grajauskas L. Song X. Gawryluk J. Lakhani B. (2022). Increased myelination plays a central role in white matter neuroplasticity. *NeuroImage* 263:119644. 10.1016/J.NEUROIMAGE.2022.119644 36170952

[B21] Lee J. Hyun J. Lee J. Choi E. Shin H. Min K. (2021). So you want to image myelin using MRI: an overview and practical guide for myelin water imaging. *J. Magn. Reson. Imaging* 53 360–373. 10.1002/JMRI.27059 32009271

[B22] Maleki S. Hendrikse J. Chye Y. Caeyenberghs K. Coxon J. Oldham S. (2022). Associations of cardiorespiratory fitness and exercise with brain white matter in healthy adults: a systematic review and meta-analysis. *Brain Imaging Behav.* 16 2402–2425. 10.1007/S11682-022-00693-Y 35773556 PMC9581839

[B23] McKenzie I. Ohayon D. Li H. de F. J. Emery B. Tohyama K. (2014). Motor skill learning requires active central myelination. *Science* 346 318–322. 10.1126/SCIENCE.1254960 25324381 PMC6324726

[B24] Mendez Colmenares A. Voss M. Fanning J. Salerno E. Gothe N. Thomas M. (2021). White matter plasticity in healthy older adults: the effects of aerobic exercise. *NeuroImage* 239:118305. 10.1016/J.NEUROIMAGE.2021.118305 34174392

[B25] Metzler-Baddeley C. Cantera J. Coulthard E. Rosser A. Jones D. Baddeley R. (2014). Improved executive function and callosal white matter microstructure after rhythm exercise in Huntington’s disease. *J. Huntington’s Dis.* 3 273–283. 10.3233/JHD-140113 25300331 PMC7613440

[B26] Naumburg E. Strömberg B. Kieler H. (2012). Prenatal characteristics of infants with a neuronal migration disorder: a national-based study. *Int. J. Pediat.* 2012:541892. 10.1155/2012/541892 22548087 PMC3324140

[B27] Paquola C. Hong S. (2023). The potential of myelin-sensitive imaging: redefining spatiotemporal patterns of Myeloarchitecture. *Biolo. Psychiatry* 93 442–454. 10.1016/J.BIOPSYCH.2022.08.031 36481065

[B28] Renke M. Marcinkowska A. Kujach S. Winklewski P. (2022). A systematic review of the impact of physical exercise-induced increased resting cerebral blood flow on cognitive functions. *Front. Aging Neurosci.* 14:803332. 10.3389/fnagi.2022.803332 35237146 PMC8882971

[B29] Rowley C. Bock N. Deichmann R. Engeroff T. Hattingen E. Hellweg R. (2020). Exercise and microstructural changes in the motor cortex of older adults. *Eur. J. Neurosci.* 51 1711–1722. 10.1111/EJN.14585 31593327

[B30] Saab A. Nave K. (2017). Myelin dynamics: protecting and shaping neuronal functions. *Curr. Opin. Neurobiol.* 47 104–112. 10.1016/J.CONB.2017.09.013 29065345

[B31] Sandrone S. Aiello M. Cavaliere C. Thiebaut de Schotten M. Reimann K. Troakes C. (2023). Mapping myelin in white matter with T1-weighted/T2-weighted maps: discrepancy with histology and other myelin MRI measures. *Brain Struct. Funct.* 228 525–535. 10.1007/S00429-022-02600-Z 36692695 PMC9944377

[B32] Shao X. Luo D. Zhou Y. Xiao Z. Wu J. Tan L. (2022). Myeloarchitectonic plasticity in elite golf players’ brains. *Hum. Brain Mapp.* 43 3461–3468. 10.1002/HBM.25860 35420729 PMC9248307

[B33] Singh T. D. Fugate J. E. Rabinstein A. A. (2014). Central pontine and extrapontine myelinolysis: a systematic review. *Eur. J. Neurol.* 21 1443–1450. 10.1111/ENE.12571 25220878

[B34] Sui Y. V. Masurkar A. V. Rusinek H. Reisberg B. Lazar M. (2022). Cortical myelin profile variations in healthy aging brain: a T1w/T2w ratio study. *NeuroImage* 264:119743. 10.1016/J.NEUROIMAGE.2022.119743 36368498 PMC9904172

[B35] Tobiume M. Iha N. Miyahira A. Kariya S. (2022). A 44-year-old alcohol-dependent man who recovered from central pontine myelinolysis with supportive physical therapy. *Am. J. Case Rep.* 23:e937389. 10.12659/AJCR.937389 36081331 PMC9472294

[B36] van der Weijden C. Biondetti E. Gutmann I. Dijkstra H. McKerchar R. de Paula Faria D. (2023). Quantitative myelin imaging with MRI and PET: an overview of techniques and their validation status. *Brain* 146 1243–1266. 10.1093/BRAIN/AWAC436 36408715 PMC10115240

[B37] van der Weijden C. García D. Borra R. Thurner P. Meilof J. van Laar P. (2021). Myelin quantification with MRI: a systematic review of accuracy and reproducibility. *NeuroImage* 226:117561. 10.1016/J.NEUROIMAGE.2020.117561 33189927

[B38] Winklewski P. Sabisz A. Naumczyk P. Jodzio K. Szurowska E. Szarmach A. (2018). Understanding the physiopathology behind axial and radial diffusivity changes-what do we know? *Front. Neurol.* 9:92. 10.3389/fneur.2018.00092 29535676 PMC5835085

[B39] Zhou H. Liu Z. Liu X. Chen Q. (2016). Umbilical cord-derived mesenchymal stem cell transplantation combined with hyperbaric oxygen treatment for repair of traumatic brain injury. *Neural Regen. Res.* 11 107–113. 10.4103/1673-5374.175054 26981097 PMC4774201

[B40] Zhou Z. Li Q. Liao C. Cao X. (2023). Optimized three-dimensional ultrashort echo time: magnetic resonance fingerprinting for myelin tissue fraction mapping. *Hum. Brain Mapp.* 44 2209–2223. 10.1002/HBM.26203 36629336 PMC10028641

